# The prophylactic effects of monoclonal antibodies targeting the cell wall Pmt4 protein epitopes of *Candida albicans* in a murine model of invasive candidiasis

**DOI:** 10.3389/fmicb.2022.992275

**Published:** 2022-08-23

**Authors:** Xiaojuan Wang, Peng Liu, Yuanying Jiang, Bing Han, Lan Yan

**Affiliations:** ^1^School of Pharmacy, Naval Medical University, Shanghai, China; ^2^Department of Pharmacy, Minhang Hospital, Fudan University, Shanghai, China; ^3^Department of Gastroenterology, Institute for Regenerative Medicine, Shanghai East Hospital, Tongji University School of Medicine, Shanghai, China; ^4^Department of Pharmacology, Shanghai Tenth People’s Hospital, Tongji University School of Medicine, Shanghai, China

**Keywords:** *Candida albicans*, monoclonal antibodies, anti-Candida mAbs, invasive candidiasis, *PMT4*

## Abstract

*Candida albicans* (*C. albicans*) is the most prevalent opportunistic human pathogen, accounting for approximately half of all clinical cases of candidemia. Resistance to the existing antifungal drugs is a major challenge in clinical therapy, necessitating the development and identification of novel therapeutic agents and potential treatment strategies. Monoclonal antibody-based immunotherapy represents a promising therapeutic strategy against disseminated candidiasis. Protein mannosyltransferase (Pmt4) encodes mannosyltransferases initiating O-mannosylation of secretory proteins and is essential for cell wall composition and virulence of *C. albicans*. Therefore, the Pmt4 protein of *C. albicans* is an attractive target for the discovery of alternative antibody agents against invasive *C. albicans* infections. In the present study, we found that monoclonal antibodies (mAbs) C12 and C346 specifically targeted the recombinant protein mannosyltransferase 4 (rPmt4p) of *C. albicans*. These mAbs were produced and secreted by hybridoma cells isolated from the spleen of mice that were initially immunized with the purified rPmt4p to generate IgG antibodies. The mAbs C12 and C346 exhibited high affinity to *C. albicans* whole cells. Remarkably, these mAbs reduced the fungal burden, alleviated inflammation in the kidneys, and prolonged the survival rate significantly in the murine model of systemic candidiasis. Moreover, they could activate macrophage opsonophagocytic killing and neutrophil killing of *C. albicans* strain *in vitro*. These results suggested that anti-rPmt4p mAbs may provide immunotherapeutic interventions against disseminated candidiasis *via* opsonophagocytosis and opsonic killing activity. Our findings provide evidence for mAbs as a therapeutic option for the treatment of invasive candidiasis.

## Introduction

Invasive fungal infections (IFI) contribute to significant annual morbidity and mortality on a global scale. Approximately 1.7 million annual lethality is attributed to lethal invasive fungal infections, which is comparable to those due to tuberculosis or AIDS, and more than those owing to malaria, breast tumor, or prostate cancer (Brown et al., 2012; Kainz et al., 2020). Strikingly, *Candida* species are the most common causes of severe fungal infections and the fourth leading cause of healthcare-associated infections in the United States (Ostrosky-Zeichner et al., 2010). Among them, *Candida albicans* is the most prevalent opportunistic fungal species, resulting in both superficial mucosal and invasive infections, including those of internal organs and candidemia (Kim and Sudbery, 2011). The annual incidence of invasive *C. albicans* infections has increased notably in immunocompromised patients suffering from malignant tumors, solid organ transplantation, or AIDS (Gow et al., 2011; Bongomin et al., 2017; Pappas et al., 2018). Furthermore, the mortality rate associated with systemic candidiasis is reportedly higher than 30% (Wisplinghoff et al., 2014). At present, the arsenal of antifungal drugs used for systemic candidiasis, including fluconazole, amphotericin B, caspofungin, and 5-flucytosine, is limited. The effective treatment of systemic infections is hindered by the emergence of resistance to currently used antifungal drugs and long-term treatment regimens (Ruggero and Topal, 2014; Lee et al., 2021). Considering the side effects, drug–drug interactions and resistance to the limited number of antifungal drugs, new therapeutic strategies against lethal fungal infections are needed (Arastehfar et al., 2020).

Antibody represents a critical component of the adaptive immune responses and is a key weapon to eradicate microbial infections (Boniche et al., 2020; Ulrich and Ebel, 2020). Targeting specific molecular targets in bacteria and viruses using monoclonal antibodies can overcome the limitations of small-molecule drugs (Zurawski and McLendon, 2020). Nowadays, it has been increasingly appreciated that antibodies are important for the effective elimination of fungal infections. Remarkably, in a pioneering study, robust antibodies responding to specific proteins of *C. albicans* were found to be generated in systemic candidiasis recovered patients, while no, minimal, or waning immune responses were exhibited in those who succumbed to these infections (Matthews et al., 1987). Therefore, passive immunization in severely immunocompromised patients with monoclonal antibodies has the potential to directly combat fungal pathogens and/or activate the residual antifungal immune responses. Unfortunately, although a few mAbs show modest efficacy in the murine model of systemic *C. albicans* infection, no antifungal mAbs are currently available for use in routine clinical practice (Lee et al., 2011; Rudkin et al., 2018; Antoran et al., 2020; Shukla et al., 2021).

The fungal cell wall maintains its shape and plays an important role in hyphal growth, adhesion, and invasion (Chaffin, 2008; Gow et al., 2011; Hiller et al., 2011; Gow and Hube, 2012; Arita et al., 2022). Concurrently, it is crucial for protecting fungi from environmental stress and simultaneously mediating the fungus-host interactions (Mckenzie et al., 2010; Gow and Hube, 2012; Gow et al., 2017). Several studies have revealed that the molecular composition and the expression of the cell wall components change in response to growth pressures, including alterations in the carbon source, iron restriction, hypoxia, and exposure to antifungal agents, indicating that these proteins may either be up- or down-regulated *in vivo* during infections (Ruiz-Herrera et al., 2006). Furthermore, cell wall proteins, as essential components of fungi, represent ideal targets for developing antifungal vaccines and antibodies (Boniche et al., 2020; Ibe and Munro, 2021). Of note, protein mannosyltransferase (Pmt4), encoding mannosyltransferases initiating O-mannosylation of secretory proteins, is one of the five members of the PMT gene family of *C. albicans*, and is critically involved in cell wall composition and virulence of *C. albicans* (Prill et al., 2005; Lengeler et al., 2008). In our previous study, vaccination with the recombinant mannosyltransferase 4 (rPmt4p) exhibited a significant protective effect in mice with invasive *C. albicans* infection. Specifically, the rPmt4p vaccine reduced mortality rate and activated both humoral and cellular immune responses (Wang et al., 2015a).

Herein, we isolated and prepared several hybridomas from the mice vaccinated with different peptides of rPmt4p. These hybridomas produced a panel of monoclonal antibodies (mAbs), displaying a range of specific binding profiles to rPmt4p. After measurements of the specificity and affinity of their binding with *C. albicans* whole cells *via* enzyme-linked immunosorbent assay (ELISA), two specific mAbs targeting rPmt4p, C12 and C346, were selected for further analysis. We focused on the potential prophylactic value of mAbs C12 and C346 in a disseminated candidiasis murine model. The mAbs significantly increased the survival rates in these fungi-infected mice and attenuated kidney damage. Furthermore, mAbs C12 and C346 enhanced the macrophage opsonophagocytic activity and neutrophil killing effects against the *C. albicans* strain *in vitro*. Our findings highlight the prophylactic value of mAbs in the treatment of disseminated candidiasis and provide an effective antibody-based therapeutic option against systemic *C. albicans* infection.

## Materials and methods

### Animals

Female C57BL/6 and BALB/c mice aged 6–8 weeks and weighing 18–22 g were procured from SLAC Laboratory Animal Co., Ltd. (Shanghai, China). All animals were housed with free access to tap water and rodent chow in a temperature-controlled animal facility with a 12-h light/dark cycle.

### *Candida albicans* strains and culture conditions

*C. albicans* SC5314 was provided by Dr. William A. Fonzi (Georgetown University, Washington, D.C., United States). *C. albicans* SC5314 were thawed from glycerol stocks stored at −80°C, plated onto SDA plates (4% dextrose, 1.8% agar, and 1% peptone), and grown in YPD broth (2% glucose, 2% peptone, and 1% yeast extract) at 30°C. For hyphal growth, exponentially growing *C. albicans* yeast cells were washed in phosphate-buffered saline (PBS) buffer and cultured in Spider or Lee’s liquid medium for 3 h at 37°C. RPMI1640 medium was used for biofilm formation assays.

### Production of mAbs

The mAbs were prepared by the Abmart Antibody Production Company (Abmart, Shanghai, China). Briefly, based on the amino acid sequence, physicochemical properties, secondary structure, and antigenicity of Pmt4p of *C. albicans*, B cell epitopes were predicted. According to the results, several specific peptides were synthesized and used to subcutaneously immunize BALB/c mice. The antibody titers in sera of the vaccinated mice were determined by indirect enzyme-linked immunosorbent assay (ELISA). Mice with high antibody titers were selected for monoclonal antibody preparation. The spleen was removed and cells were dispersed to obtain a single cell suspension. The immunized mice spleen and myeloma SP2/0 cells were fused to produce hybridoma cell lines. Positive hybridomas capable of secreting mAbs against the corresponding peptides were identified by ELISA and subjected to cloning and subcloning by the limiting dilution method. The positive hybridoma cells were injected intraperitoneally into BALB/c mice to obtain ascites fluid. The mAbs were collected and purified by the protein-G affinity column (Abmart, Shanghai, China).

### ELISA

ELISA was performed in 96-well plates to detect antibody titers (Costar, United States). After overnight culture, *C. albicans* cells were washed thrice with PBS and subsequently resuspended in RPMI 1640 medium to a final concentration of 1 × 10^6^ cells/mL. To a 96-well plate, 100 μl of cell suspensions and coating buffer (0.1 M NaHCO_3_ and 0.1 M Na_2_CO_3_; pH 9.6) were added. Following overnight incubation at 4°C, the wells were washed five times with PBS containing 0.05% Tween 20 (PBST); blocked with 200 μl of blocking solution (0.1% BSA in PBS), and incubated for 2 h at 37°C. After washing thrice with PBST, 100 μl of purified mAb in blocking buffer was added per well and the plates were incubated at 37°C for 2 h. PBST and IgG were the negative controls. Wells were washed thrice before the addition of horseradish peroxidase (HRP)-conjugated goat anti-mouse IgG (H + L; KPL) at 1:5000 dilution and incubated at 37°C for 1 h. Following a final round of washing, 3,3′,5,5′-tetramethylbenzidine (TMB, Sigma) substrate (100 μl) was added. The plates were incubated at room temperature for 10 min in the dark, followed by the addition of 50 μl of 2 M H_2_SO_4_ to terminate the reactions. The absorbance of each well was measured at 450 nm on a plate reader (Multiskan MK3, Finland). GraphPad Prism 9 was used to generate concentration-response curves for half-maximal effective concentration (EC_50_) determination.

### Murine model of systemic candidiasis

To assess the antifungal effects of mAbs *in vivo*, survival rate and kidney histopathological assessments were determined in the murine model of disseminated candidiasis. *C. albicans* SC5314 strain was harvested, resuspended in saline, and counted. BALB/c mice were challenged with 1 × 10^5^ *C. albicans* SC5314 through tail vein injection. At 2 h prior to challenge, saline, IgG control, 1, 2, 3, or 4 mg/kg of mAbs were administered to the corresponding mice by intravenous injection. The mice were monitored for 40 days post-inoculation. The kidneys from 6 mice in each group were aseptically harvested, weighed, and homogenized in PBS on day 2 post-infection. The serial dilutions of each group were plated on SDA plates and incubated overnight at 30°C. The colony-forming units (CFU) were counted. The fungal burdens of kidneys were computed as a ratio of CFU/g of the organ. For histopathological assessment, the kidneys were fixed with 10% neutral formalin, dehydrated in graded alcohol solutions, embedded in paraffin, and stained with hematoxylin and eosin (H&E) or periodic acid-Schiff (PAS) solutions.

### Determination of macrophage phagocytosis and neutrophil killing

Macrophage phagocytosis and neutrophil killing assays were conducted as reported previously with slight modifications (Zhang et al., 2016; Chen et al., 2020). Briefly, from healthy 6-8-week-old C57BL/6 mice, peritoneal macrophages and neutrophils stimulated by thioglycollate were isolated. Following three washes with PBS, the concentration of overnight cultured *C. albicans* SC5314 was adjusted to 1 × 10^5^ cells/mL. For the macrophage phagocytosis killing assay, *C. albicans* SC5314 was co-cultured with peritoneal macrophages at MOI = 0.4 in the presence of 50, 100, or 150 μg/ml mAbs for 1 h at 37°C, followed by washing thrice in PBS. The mixture was plated on YPD agar for 48 h at 30°C and the surviving *C. albicans* strain was counted.

For the neutrophil killing assay, overnight cultured *C. albicans* SC5314 was incubated with indicated concentrations of mAbs for 1 h at 37°C. After washing with PBS, the concentration of *C. albicans* was adjusted to 1 × 10^5^ cells/mL. Neutrophils were co-cultured with mAb-treated *C. albicans* strain at MOI = 0.05 for 1 h at 4°C before further incubation for another 1 h at 37°C. The mixture was plated on YPD agar at 30°C for 48 h and the surviving *C. albicans* strain was counted. The killing rates were finally calculated using the following formula:


$$\left(1-\frac{\begin{array}{l} \mathrm{colonies of}\;C.\mathrm{albicans}\;\mathrm{incubated}\;\\ \mathrm{with macrophages or neutrophils} \end{array}}{\begin{array}{l} \mathrm{colonies of}\;C.\mathrm{albicans}\;\mathrm{incubated}\;\\ \mathrm{without macrophages or neutrophils} \end{array}}\right)\times 100\%$$

### Hyphal growth assay

To evaluate the effects of mAbs on hyphal growth, *C. albicans* SC5314 cultured overnight was washed thrice in PBS and resuspended in Spider or Lee’s medium at 1 × 10^6^ cells/ml. To each well of a 96-well plate, 100 μl of the suspension was added. A final concentration of 100, 150, or 200 μg/ml of mAbs was added. Saline and IgG were the controls. The samples were incubated at 37°C for 3 h, and hyphal morphologies of the *C. albicans* strain were photographed under the EVOS inverted microscope (AMG, United States).

### Biofilm formation assay

The *in vitro* biofilm formation assay was conducted following an established protocol with minor modifications (Pierce et al., 2008). Briefly, *C. albicans* SC5314 strain was grown in YPD overnight at 30°C. After washing thrice with PBS, the strain was diluted in RPMI 1640 medium till optical density (OD) was 0.1. To a 96-well plate, 100 μl of the diluted *C. albicans* strain suspension was added to each well, and allowed to adhere for 90 min at 37°C. Each well was gently washed with PBS to remove non-adherent cells. Fresh RPMI1640 medium (100 μl) with the indicated concentrations of mAbs was added to the corresponding wells and incubated at 37°C for 24 h. IgG and saline were the negative controls. The inhibition of biofilm formation by mAbs was assessed by the previously reported 2,3-bis-(2-methoxy-4-nitro-5-sulfophenyl)-2H-tetrazolium-5-carboxanilide (XTT, Sigma) reduction assay (Ramage et al., 2001).

### Statistical analysis

GraphPad Prism 9 software was used to analyze the statistical significance of all data. At least three independent replicates were conducted for all experiments unless otherwise stated and *p* < 0.05 was considered statistically significant. Data analysis of mice survival was performed by the log-rank test. For parametric data, the unpaired two-tailed Student’s *t*-test was used for between-group comparison and one-way analysis of variance (ANOVA) for multiple-group comparison. For nonparametric data, the nonparametric *t*-test or analysis of variance (ANOVA) was applied.

## Results

### Anti-rPmt4p mAbs C12 and C346 bind to *Candida albicans* with high specificity and affinity

**Figure 1:**
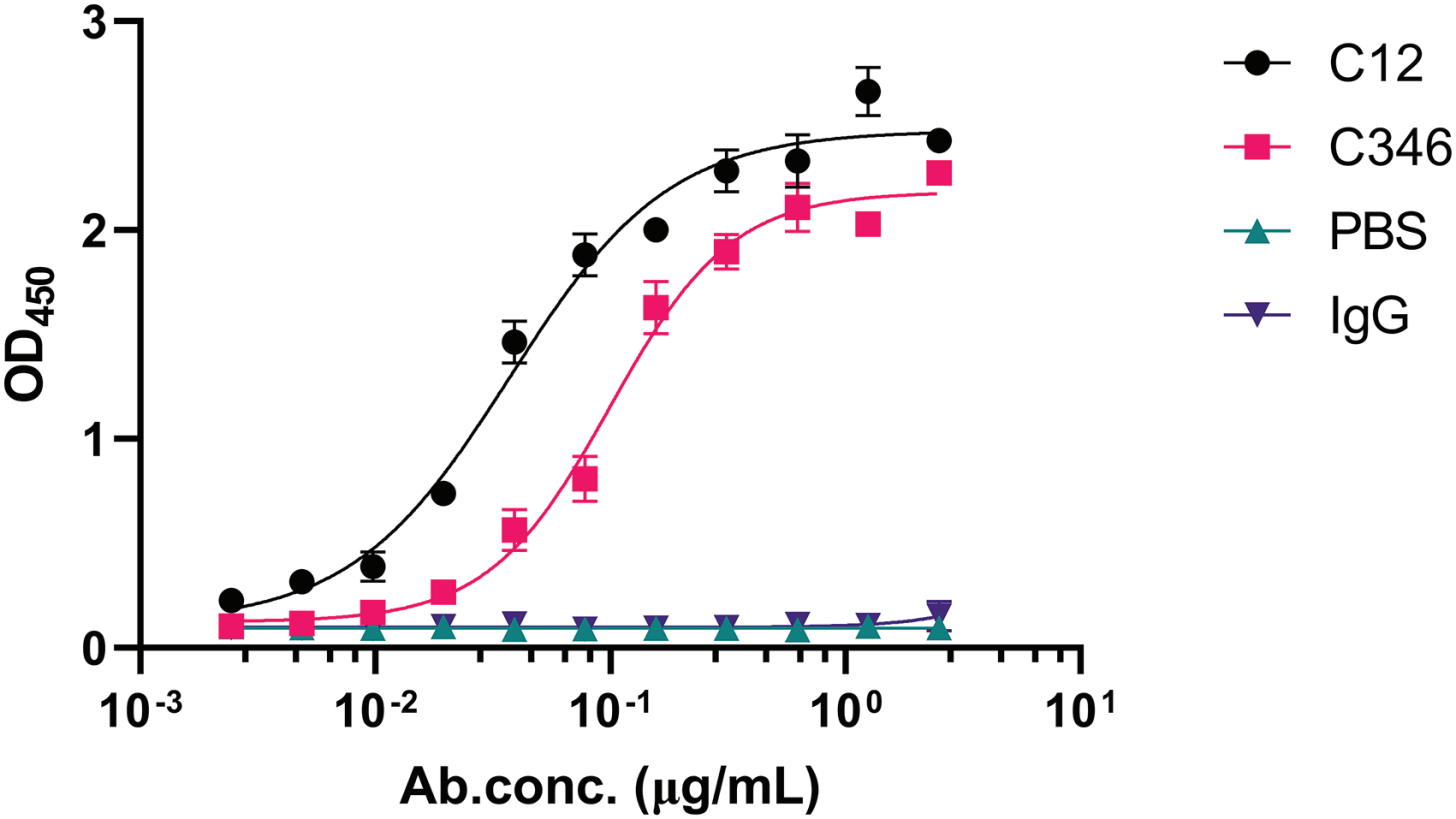
ELISA for detecting the binding ability of the purified anti-rPmt4p mAbs to *C. albicans* whole cells. Data are expressed as means ± SEM (*n* = 3).

The hybridomas were preliminarily screened by ELISA to detect their capability of binding to the recombinant *C. albicans* Pmt4p. A total of 22 positive hybridoma cells with an ELISA titer higher than 100 K were obtained. Among them, based on their binding abilities, anti-rPmt4p mAbs C12 and C346, corresponding to the peptide antigens, HVPGSNPKKEKN and LESPLAAHSKPV, respectively, were further selected by ELISA screening against *C. albicans* whole cells. Anti-rPmt4p mAb C12 showed strong binding to *C. albicans* whole cells with EC_50_ value between 30.70 and 45.27 ng/ml (Figure 1). Anti-rPmt4p mAb C346 bound to *C. albicans* whole cells with relatively lower affinity and the EC_50_ value ranged from 87.64 to 113.40 ng/ml. Therefore, mAbs showing specific binding to *C. albicans* cells were generated.

### Anti-rPmt4p mAbs C12 and C346 protect mice against disseminated candidiasis

**Figure 2:**
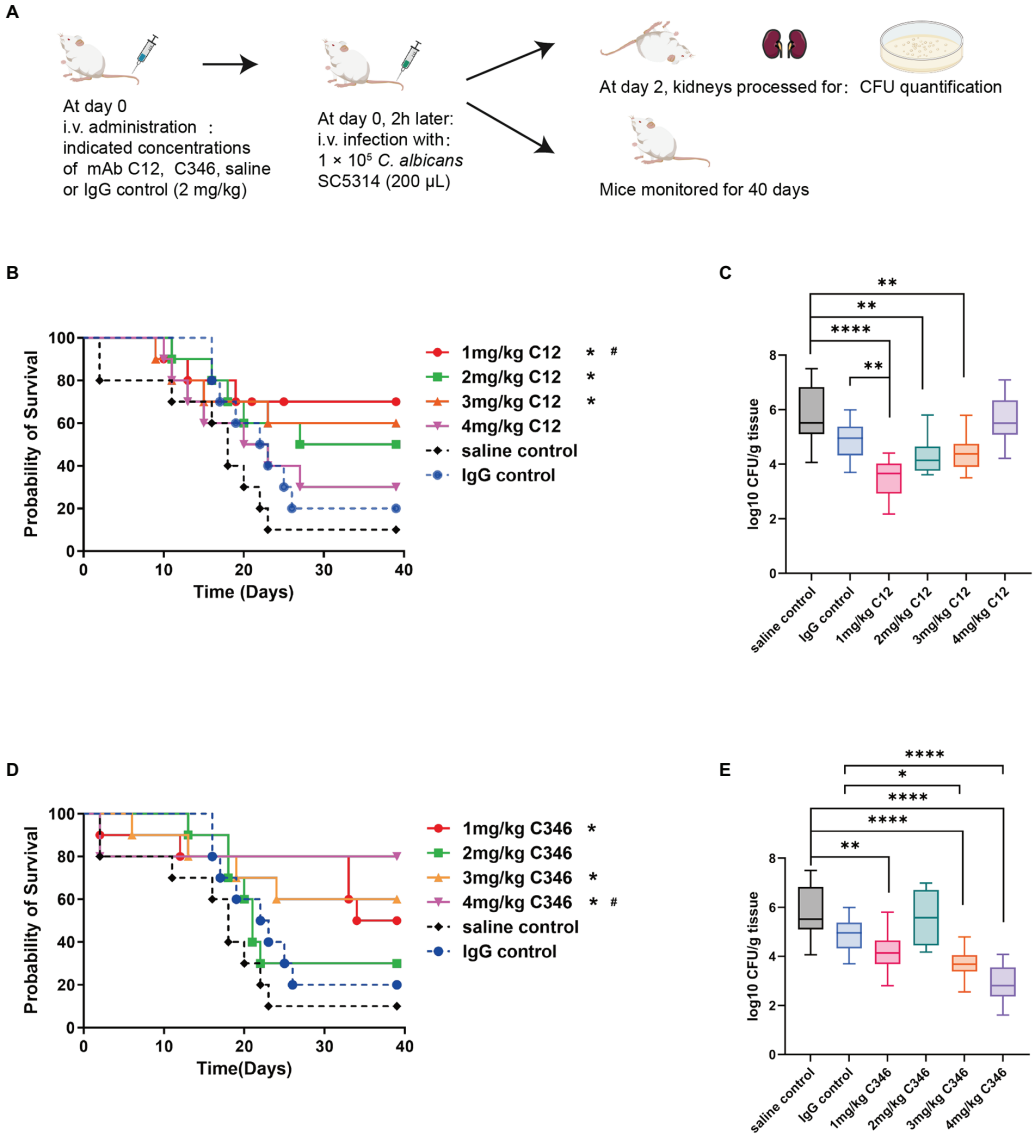
The *in vivo* antifungal efficacy of anti-rPmt4p mAbs in the murine model of disseminated candidiasis. **(A)** Schematic process of pre-treatment with mAb C12 or mAb C346 in the murine model of disseminated candidiasis. **(B,C)** Mice were prophylactically administrated 1, 2, 3, 4 mg/kg of mAb C12, saline, or 2 mg/kg of IgG. Two hours later, mice were challenged with 1 × 10^5^ *C. albicans* SC5314 cells/mouse *via* the lateral tail vein. **(B)** The Kaplan–Meier survival curves of mice monitored for 40 days post-inoculation (*n* = 10 per group). **(C)** Quantification of fungal burden in the kidneys from mAb C12 treated mice on day 2 post-infection (n = 6 per group). **(D,E)** Mice were prophylactically administrated 1, 2, 3, 4 mg/kg of mAb C346, saline, or 2 mg/kg of IgG. Two hours later, mice were challenged with 1 × 10^5^ *C. albicans* SC5314 cells/mouse *via* the lateral tail vein. **(D)** The Kaplan–Meier survival curves of mice monitored for 40 days post-inoculation (*n* = 10 per group). **(E)** Quantification of fungal burden in the kidneys from mAb C346 treated mice on day 2 post-infection (*n* = 6 per group). ^*^, *p* < 0.05 vs. saline control group; ^#^, *p* < 0.05 vs. IgG control group (**B,D**; Log-rank test). ^*^, *p* < 0.05; ^**^, *p* < 0.01; ^****^, *p* < 0.0001 (**C,E**; Nonparametric One-way ANOVA).

To investigate the protective efficacy of the antibodies *in vivo*, the survival rate of mice pretreated with mAb C12 or mAb C346 in the disseminated candidiasis mice was assessed (Figure 2A). The survival rates (at the end of the monitoring period) in mice administrated mAb C12 at 1, 2, and 3 mg/kg were 70, 50, and 60%, respectively. The lower dose of mAb C12 1 mg/kg significantly increased the 40-day survival rate of mice from 10 to 70% relative to the saline group. Moreover, the protective effects were superior to those in the IgG group with a survival rate of 20% (Figure 2B). The median survival time in the saline and IgG groups was 22 and 22.5 days, respectively. 2 mg/kg of mAb C12 significantly enhanced the median survival time to 33 days. In particular, 1 mg/kg of mAb C12 prevented death in 70% mice (*p* < 0.05). Nevertheless, the protective effects of mAbs *in vivo* were not induced in a dose-dependent manner. Additionally, disease progression was measured based on the fungal burden in the kidneys. Prophylactic administration of 1, 2, and 3 mg/kg of mAb C12 significantly reduced the fungal burden in the kidneys compared to the saline control, consistent with the results of the survival analysis (Figure 2C). Compared with the IgG group, pre-administration of mAb C12 (1 mg/kg) also significantly reduced the fungal burden in kidneys (*p* < 0.01).

Similarly, the mAb C346 exerted protective effects against disseminated candidiasis (Figure 2D). Relative to the saline group, administration of 1, 3, and 4 mg/kg of mAb C346 improved the survival rates of mice from 10 to 50, 60, and 80%, respectively. Furthermore, significant survival extension upon administration of 4 mg/kg mAb C346 relative to IgG was observed (*p* < 0.05). Likewise, mAb C346 administration (3 and 4 mg/kg) reduced the fungal burden in kidneys relative to those in the saline and IgG groups significantly (Figure 2E). These results suggested that the anti-rPmt4p mAbs C12 and C346 provided prophylactic protection in the murine model of the disseminated candidiasis.

### mAbs attenuate the damage caused by the *Candida albicans* strain and reduce inflammation in the kidneys

**Figure 3:**
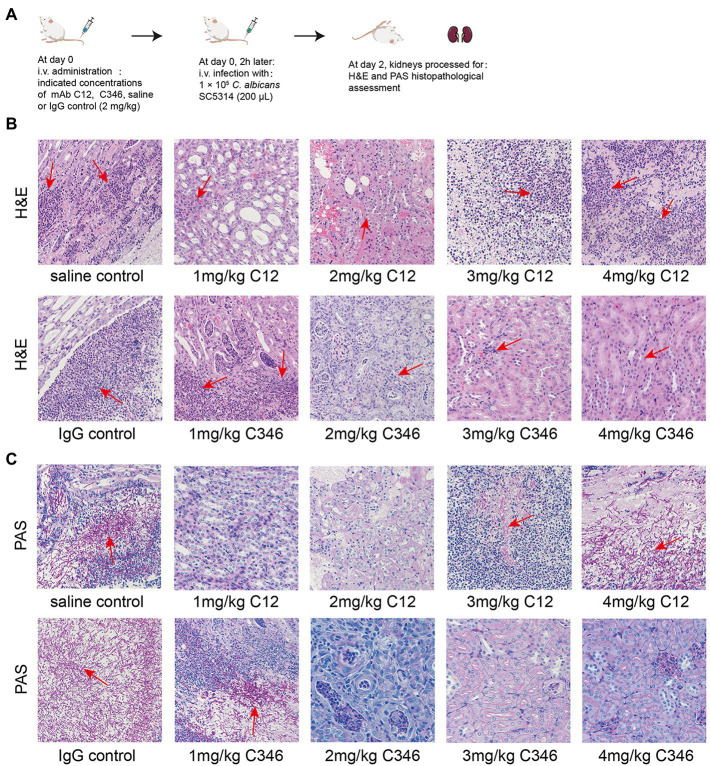
The effects of pre-treatment with mAb C12 or mAb C346 on renal histopathological damage in the murine model of disseminated candidiasis. **(A)** Schematic process of pretreatment with mAb C12 or mAb C346 in the murine model of disseminated candidiasis. The kidneys were dissected on day 2 post-infection with *C. albicans* SC5314. **(B,C)** Representative histopathological assessments of the kidneys by H&E staining for inflammatory cell influx and extent of tissue necrosis **(B)** and PAS staining for *C. albicans* strain **(C)**. Arrows indicate inflammatory cell influx and tissue necrosis (H&E staining), and *C. albicans* filaments in the tissues (PAS staining). The representative results of three independent experiments are shown. Magnification 200 ×.

Furthermore, the histopathological status of kidneys challenged by *C. albicans* was assessed by H&E and PAS staining assays. As shown in Figure 3B, H&E staining revealed massive renal medullary necrosis and inflammatory cell infiltration in the saline and IgG groups. PAS staining showed colonization by hyphae and pseudohyphae in the renal medulla and pelvis in both the saline and IgG groups (Figure 3C). In contrast, kidney tissue necrosis and inflammatory responses were remarkably ameliorated and no obvious *C. albicans* colonization or infection lesion was found upon treatment with mAb C12 (1 mg/kg) or mAb C346 (4 mg/kg), consistent with the results of survival and kidney fungal burden analyses.

These results suggested that mAbs C12 and C346 have promising potential antifungal activity *in vivo* as they exerted protective effects against the invasion of *C. albicans* strain in the kidneys and prolonged the survival in the murine model of disseminated candidiasis.

### Anti-rPmt4p mAbs C12 and C346 promote macrophage opsonophagocytic and neutrophil killing activity against the *Candida albicans* strain

**Figure 4:**
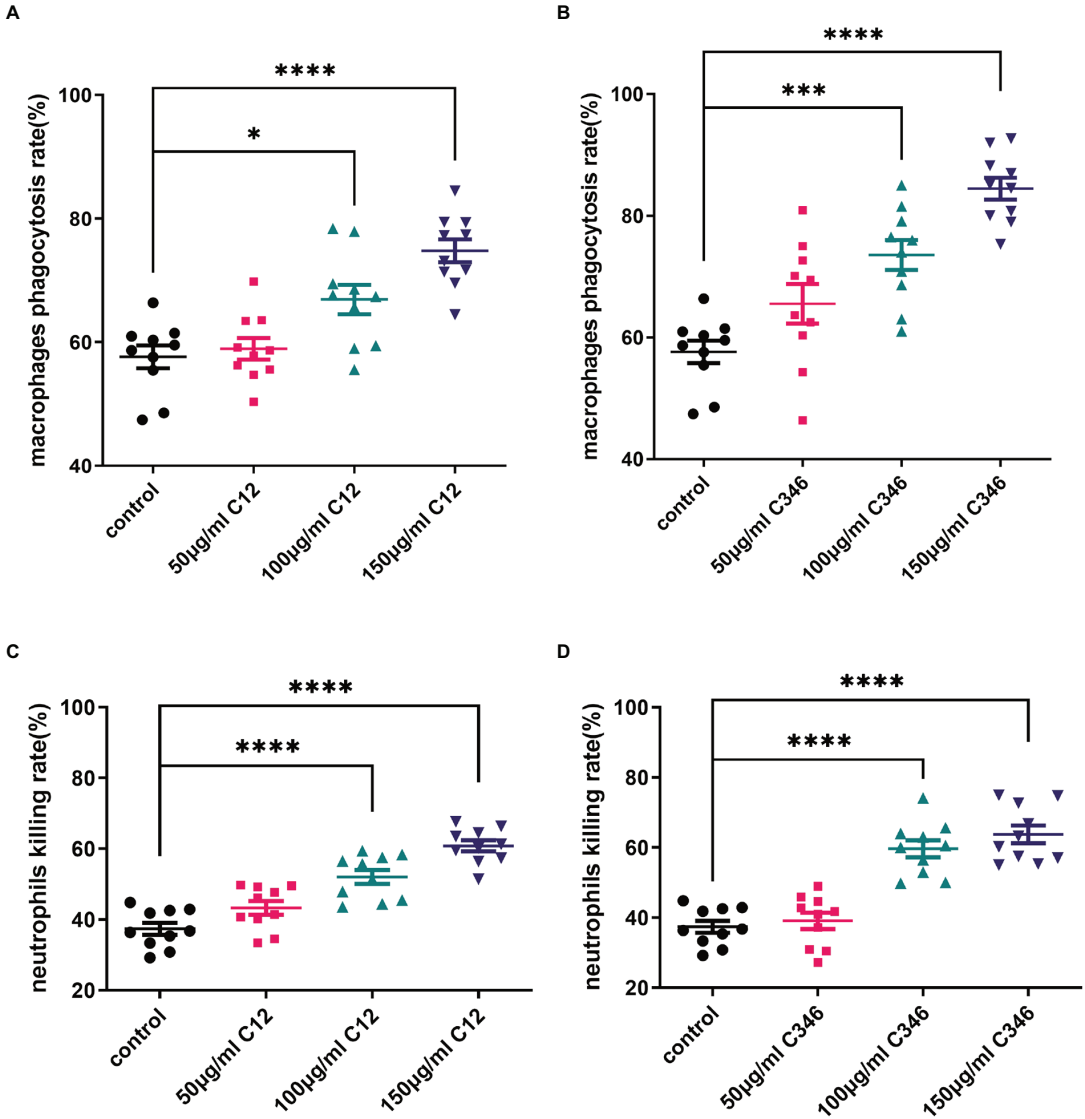
The phagocytosis and killing rates of thioglycollate-elicited peritoneal macrophages and neutrophils pretreated with anti-rPmt4p antibodies against *C. albicans* SC5314. *C. albicans* SC5314 was incubated with thioglycollate-elicited peritoneal macrophages (MOI = 0.4) in the presence of indicated concentrations of mAb C12 **(A)** and mAb C346 **(B)** for 1 h at 37°C, followed by washing thrice in PBS. The suspension was plated on YPD agar at 30°C for 48 h; the colonies were counted and the phagocytosis rate was calculated. *C. albicans* SC5314 cells were co-cultured with thioglycollate-elicited peritoneal neutrophils (MOI = 0.05) with the indicated concentrations of mAb C12 **(C)** and mAb C346 **(D)** at 37°C for 1 h. The suspension was then plated on YPD agar for 48 h. *C. albicans* colonies were counted and the killing rates were calculated. Data in **(A–D)** are representative of three independent experiments. ^*^, *p* < 0.05; ^***^, *p* < 0.001; ^****^, *p* < 0.0001, one-way ANOVA.

To determine whether the mAbs contributed to the killing of *C. albicans* by regulating mAb-mediated fungal opsonophagocytosis and opsonic-killing, killing assays were performed using mice macrophages and neutrophils. As shown in Figure 4, mAbs C12 and C346 exhibited opsonophagocytosis and opsonic-killing activity in a dose-dependent manner when co-incubated with macrophages or neutrophils, and challenged by the *C. albicans* strain. Rates of phagocytosis by macrophages and neutrophil killing increased significantly when macrophages or neutrophils were pre-incubated with mAb C12 or C346 at 100 and 150 μg/ml. Taken together, mAb C12 and mAb C346 enhanced the antibody-dependent opsonophagocytosis and opsonic-killing activity of macrophages and neutrophils against the *C. albicans* strain.

### Anti-rPmt4p mAbs C12 and C346 do not inhibit hyphal growth or biofilm formation

**Figure 5:**
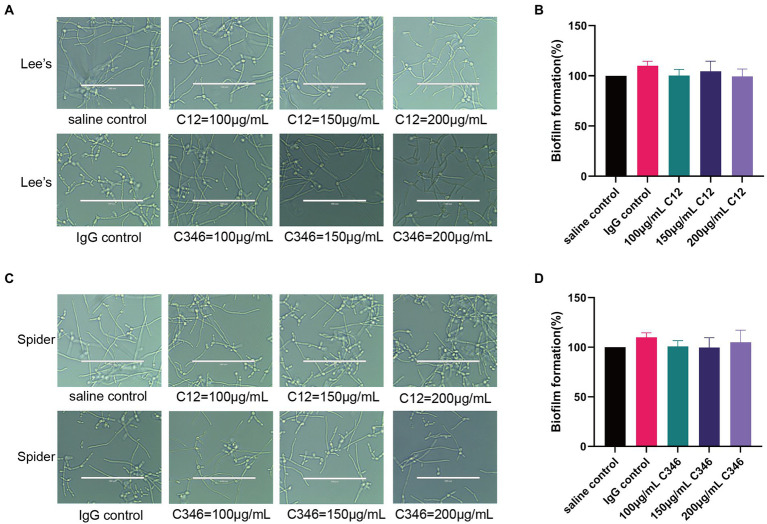
No obvious inhibitory effects of anti-rPmt4p mAbs C12 and C346 on hyphal growth or biofilm formation. Exponentially growing *C. albicans* SC5314 cells were transferred to Lee’s liquid medium **(A)** or Spider medium **(C)**, followed by co-incubation with different concentrations of mAbs. The cellular morphologies were photographed after incubation at 37°C for 3 h. Scale bar = 100 μm for **(A,C)**. Exponentially growing *C. albicans* SC5314 cells were transferred to RPMI1640 liquid medium in a 96-well plate for 90 min, followed by addition and incubation for 24 h with mAbs, C12 **(B)** or C346 **(D)**. Biofilm formation was evaluated by the XTT reduction assay. The results are presented as the percentage compared to the biofilm formed in the saline control group. Data in **(A,C)** are representative of three independent experiments. Data in **(B,D)** are expressed as means ± SD (*n* = 3).

To investigate whether mAb C12 or C346 could inhibit hyphal growth and biofilm formation, *C. albicans* strain SC5314 was cultured with or without the mAbs in Lee’s or Spider medium. As shown in Figure 5, normal hyphae and biofilm formation were observed upon mAb treatment at indicated concentrations in each medium, relative to the control group. Thus, the mAbs did not inhibit hyphal growth or biofilm formation. Therefore, we reasonably speculate that anti-rPmt4p mAbs’ antifungal effects may be through antibody-dependent opsonophagocytosis and opsonic-killing rather than direct inhibition of hyphal or biofilm formation.

## Discussion

The treatment and management of invasive fungal infections are compromised by long-term treatment regimens and antifungal drug resistance in many fungal genera (Pappas et al., 2018). Vaccines or antibodies alone or in combination with chemotherapy can prevent post-treatment sequelae and reestablish a protective immune response (Biswas, 2021). Several studies have confirmed the immunogenicity and efficacy of vaccines using live attenuated *C. albicans* strains, purified recombinant proteins (Als1p, Sap2p, Hsp90p, Hyr1p, and Als3p), glycoconjugate vaccines (β-Glucan conjugate vaccine, β-mannan, and peptide conjugates,), and cell wall extract (β-mercaptoethanol extract) against candidiasis in animal models (Wang et al., 2015b). Among them, the rAls3p vaccine showed efficacy and safety according to a phase II clinical trial (Edwards et al., 2018). mAbs alone or their combinations are expected to show great protective potential in antifungal therapy, particularly to reduce the high morbidity and mortality in immunocompromised patients (Boniche et al., 2020). Monoclonal antibodies against fungi have been evaluated as an alternative therapeutic option against life-threatening systemic candidiasis. Indeed, several reports have validated the generation of protective antibodies as a critical aspect of recovery from infection (Pelfrene et al., 2019; Boniche et al., 2020; Ulrich and Ebel, 2020). mAbs recognize antigens that are specific to the fungi, such as polysaccharides and proteins in the fungal cell wall (β-glucan, Als3, Sap2, Hsp90, Hry1, Eno1, Utr2, and Pga31 implicated in fungal integrity, assembly, adhesion, virulence, morphogenesis, and pathogenesis) and show protection against fungal infections (De Bernardis et al., 1997; Matthews et al., 2003; Pachl et al., 2006; Rachini et al., 2007; Laforce-Nesbitt et al., 2008; Beucher et al., 2009; Coleman et al., 2009; Torosantucci et al., 2009; Rudkin et al., 2018; Matveev et al., 2019; Chen et al., 2020; Heredia et al., 2020; Leu et al., 2020). For example, antibodies associated with Als3p include mAbs C7, 3D9.3, 2G8, and scFv3 (Laforce-Nesbitt et al., 2008; Beucher et al., 2009; Coleman et al., 2009; Torosantucci et al., 2009). The mAb 2G8 provides marked protection against both systemic and mucosal candidiasis, evidenced in passive vaccination experiments in mice (Torosantucci et al., 2009), while scFv3 can suppress *C. albicans* adhesion to human cells (Laforce-Nesbitt et al., 2008). Thus, antibody neutralizing virulence factors of *C. albicans* are valuable in the treatment of candidiasis, especially in immunocompromised hosts.

Remarkably, cell wall proteins are extremely important for fungi to maintain cell morphology and pathogenicity, and adapt to the external environment. Furthermore, cell wall components can be recognized by the innate immune system, the first line of defense against fungal invasion (Sukhithasri et al., 2013). Thus, vaccines or mAb-based strategies that selectively and effectively inhibit the virulence factors have the clinical development potential. Protein mannosyltransferase 4 represents one of the PMT gene family localized to the *C. albicans* cell wall; it encodes five isoforms of protein mannosyltransferases, which initiates O-mannosylation of secretory proteins, and is essential for the maintenance of hyphal growth, virulence, and cell wall composition (Prill et al., 2005). Previously, we have confirmed that rPmt4p vaccination improves the survival rate in a murine model of disseminated candidiasis and can serve as a vaccine candidate against systemic candidiasis (Wang et al., 2015a). Herein, we prepared anti-rPmt4p mAbs to investigate whether these mAbs protected mice against disseminated candidiasis and examined the potential protective mechanisms.

Anti-rPmt4p mAbs C12 and C346 bound specifically to *C. albicans* whole cells (Figure 1). Additionally, *in vivo* protective efficacy of these mAbs against systemic candidiasis was convincingly demonstrated in the murine model of disseminated candidiasis (Figure 2). However, this beneficial effect did not show a clear dose-dependence. mAb C12, in particular, when administered at a single dose of 1 mg/kg followed by the *C. albicans* attack, conferred improved survival rates to 70% compared to the saline (10%) and IgG (20%) controls (Figure 2B). The benefits of these antibodies were also reflected by significant reductions in the fungal burden in the kidneys of mice administered 1 mg/kg, 2 mg/kg, or 3 mg/kg mAb C12 (Figure 2C). In contrast, 4 mg/kg of mAb C346 was administered as a prophylactic before the *C. albicans* challenge and showed the most significant survival benefit and reduction in fungal burden (Figures 2D,E). Simultaneously, H&E and PAS staining assays in kidneys showed that renal injury and inflammation in the antibody-pretreated group reduced significantly relative to the saline and IgG groups (Figure 3). These results revealed that mAb C12 and mAb C346 exerted protective effects against the murine model of disseminated candidiasis at appropriate dosing and increased the clearance of *C. albicans*, thereby reducing kidney damage.

Antibodies combat pathogens mostly by direct neutralization or subsequently elicit innate immune cells opsonophagocytosis and cytotoxic responses through antibodies’ Fc domains (Lu et al., 2018). Macrophages and neutrophils are the most important effector cells of the innate immune system against *C. albicans* (Kumar and Sharma, 2010; Wang, 2015; Pappas et al., 2018). The innate immune defense system is activated after invasion by *C. albicans*. The binding between the antibody and *C. albicans* strain induces innate host immune cell-mediated phagocytosis and killing (Chen et al., 2020). Previously published studies have focused on *in vivo* efficacy, whereby mAbs were either pre-incubated with *C. albicans* or administered as a prophylactic before the challenge, resulting in survival benefits and reduction in the fungal burden in various organs (Rudkin et al., 2018; Matveev et al., 2019). Therefore, mAbs are already available in the systemic circulation and can bind to the *C. albicans* strain, thus facilitating opsonophagocytosis and clearance with increased protection. The rPmt4p-specific antibodies attenuated the kidney fungal burden in the mice received prophylactic treatment, thus indicating that mAbs can bind to *C. albicans in vivo*, probably by inhibition of cell replication and/or by enhancement of macrophage recruitment and neutrophil-mediated phagocytosis and clearance. Our results depicted that mAb C12 and C346 significantly promoted the clearance of *C. albicans* cells by opsonizing macrophage phagocytic killing and neutrophil killing activity (Figure 4).

Many therapeutic mAbs exert their protective effects through direct inhibition of hyphal growth and biofilm formation (Carrano et al., 2019; Leu et al., 2020; Palliyil et al., 2022). We investigated the inhibitory effects of anti-rPmt4p antibodies on the yeast-to-hypha morphological transition and the formation of biofilm in *C. albicans*. Unexpectedly, both mAb C12 and mAb C346 did not show inhibition of hyphal growth or biofilm formation at different concentrations (Figure 5). As biofilm is developed by the hyphae, these results are consistent and reasonable. Taken together, mAb C12 and mAb C346 may exert protection by enhancing host opsonophagocytic activity instead of inhibiting hyphal growth or biofilm formation.

The present study has some limitations. First, the absence of binding to a *PMT4* knockout strain in *C. albicans* would further enhance the specificity of mAbs C12 and C346. The binding affinities of anti-rPmt4p mAbs were only assessed in *C. albicans* strain. Further investigation of the binding profiles to other pathogenic fungi is needed to be validated. Second, invasive fungal infections can also be caused by fluconazole-resistant *C. albicans*, *C. glabrata*, or *C. parapsilosis.* The protective effects of anti-rPmt4p mAbs on these systemic fungal infections need further evaluation. Third, a single dosage of anti-rPmt4p mAbs was evaluated herein. The effects of repeated treatment with anti-rPmt4p mAbs or their combination with currently used antifungal agents remain unknown. Therefore, the prophylactic and therapeutic efficacies of anti-rPmt4p mAbs in animal models and the potential clinical use of such antibodies in the future warrant further investigation.

**Figure 6:**
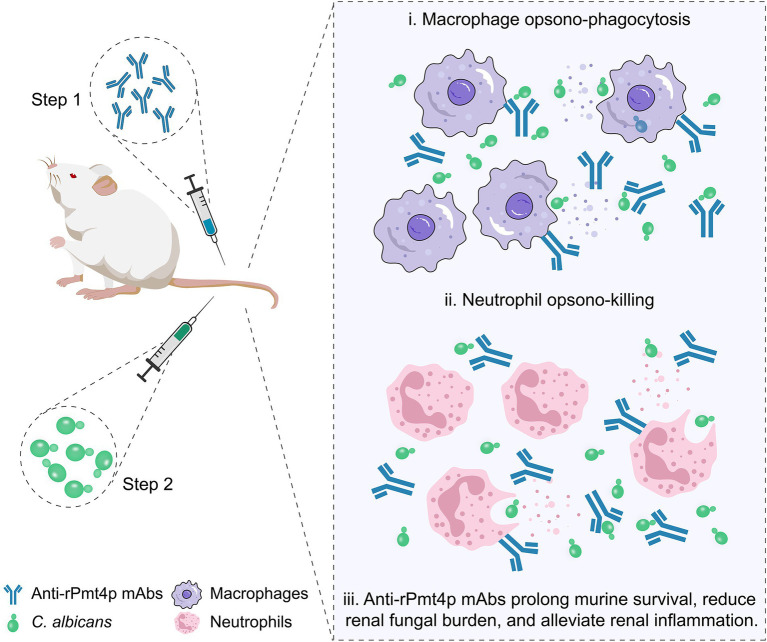
A schematic illustration of anti-rPmt4p mAbs eliciting beneficial effects in the murine model of disseminated candidiasis. The mAbs exert protective effects *via* a mechanism that involves mAb-induced macrophage opsonophagocytic (i) and neutrophil killing activities (ii). Prophylactic mAb administration prolongs the survival time, reduces the fungal burden, and alleviates renal inflammation in mice with disseminated candidiasis (iii).

In conclusion, two anti-rPmt4p mAbs were designed, produced, and shown to have prominent binding affinities to *C. albicans* whole cell. A murine model of systemic candidiasis was utilized to assess the prophylactic efficacy of the anti-rPmt4p mAbs *in vivo*. We confirmed that the mAbs exerted their protective effects through the recruitment of macrophages and neutrophils *via* antibody-mediated opsonophagocytosis and clearance (Figure 6). Thus, these findings provide new insights into anti-*C. albicans* immunotherapy and the possibility of developing potential novel antifungal therapeutic mAbs targeting the cell wall proteins.

## Data availability statement

The original contributions presented in the study are included in the article/Supplementary material, further inquiries can be directed to the corresponding authors.

## Ethics statement

The animal study was reviewed, approved, and conducted following the Animal Care Ethics guidelines with protocols approved by the Animal Care and Use Committees of Naval Medical University and Fudan University (2021JSMinhang Hospital-036).

## Author contributions

XW, PL, YJ, BH, and LY conceptualized the study design. XW and PL assessed the prophylactic value of mAbs in the murine model of disseminated candidiasis, wrote the first version of the manuscript, and conducted hyphal growth and biofilm formation analyses. XW analyzed IgG antibody’s affinity to *Candida albicans* and performed opsonophagocytosis and opsonic-killing experiments. YJ, BH, and LY supervised the study. LY revised the manuscript. All authors contributed to the article and approved the submitted version.

## Funding

This research was supported by grants from the National Natural Science Foundation of China (Nos.: 82173867, 82103095, and 82104242), the Shanghai Science and Technology Innovation Action Plan, the International Science and Technology Cooperation Project (21430713000), Shanghai Pujiang Program (21PJD0081), the Project of Shanghai Minhang District Health and Family Planning Commission (2021MW18), and Minhang District Healthcare System Program for Outstanding Young Medical Technical and Pharmacology Scholars (mwyjyx01).

## Conflict of interest

The authors declare that the research was conducted in the absence of any commercial or financial relationships that could be construed as a potential conflict of interest.

## Publisher’s note

All claims expressed in this article are solely those of the authors and do not necessarily represent those of their affiliated organizations, or those of the publisher, the editors and the reviewers. Any product that may be evaluated in this article, or claim that may be made by its manufacturer, is not guaranteed or endorsed by the publisher.
